# The Plans and Purposes of the Johns Hopkins Hospital

**Published:** 1889-08-31

**Authors:** John S. Billings

**Affiliations:** Surgeon, U.S. Army


### The Plans and Purposes of the Johns Hopkins Hospital.*

By John S. Billings, M.D., Surgeon U. S. Army.

( *Continued from page 331* )

### III.—A NURSE TRAINING SCHOOL.

A word now on the fourth object kept in view in this hospital—viz., the Training School for Nurses.

Some of you probably have had some personal experience of the difference between an educated, properly trained female nurse and one of the old-fashioned sort, but if you have not, it would take much more time than I now have to describe it. I can only say that in many cases a competent trained nurse is as important to the success of treatment as a competent doctor, and that one of the greatest difficulties in treating well-to-do patients in their own homes in this city is the want of proper nurses. Affection and zeal may do much, but they cannot take the place of knowledge, and this kind of knowledge is not to be acquired in a day or in a month. It is a work best carried out by women, though not one woman in ten is fit for it, or should undertake it. But the woman who is fit for it, who has physical health and strength, sound sense, loving kindness, patience and tact, and who has been thoroughly taught the art of nursing the sick, with all its thousand details, has the power of doing good and increasing happiness to a degree which few others possess. In a properly conducted hospital ward she is a necessity, but her field of usefulness and helpfulness is by no means limited to that. She is needed outside the hospital, in the home of the rich, to nurse and care for the sick; in the home of the poor, to teach prevention as well as nursing. To gather here such women, to have them thoroughly instructed, to furnish them with the attractive and comfortable home which they deserve, and to send them where they are most needed, with provision for their return when the work is done, is the object of the training school of this hospital.

For this purpose the trustees have provided a large and handsome building, separated from the others, and exclusively appropriated to the female nurses, where each can have her own comfortable room, and where a common parlour, library, dining-room, bathrooms, and, in short, the arrangements of a first-class hotel are provided for their use. Here also is a training-kitchen and a lecture-room to aid in the work of instruction. The intention is that when the nurse has finished her six or eight hours' tour of duty with the sick, she shall come quite away from the ward and all that pertains to it, and take her rest and recreation in a totally different atmosphere, and special effort has been made to have this home attractive and pleasant.

The fifth object which I mentioned as having been kept in view in the plan and construction of these buildings is the Dispensary. This is a large building on the north front, consisting of a large central waiting-room, surrounded by a number of smaller rooms for the use of the physicians and surgeons who are to examine and prescribe for the patients, and having bathrooms, and a small apothecaries' establishment for the issue of the medicines ordered. This building is connected with the amphitheatre by a short covered corridor, and is specially arranged with reference to teaching. It, as well as the amphitheatre, is heated by steam instead of hot water, partly because they are not in constant use, and a rapid means of warming is desired, partly for the purpose already referred to, of giving the means of experimental comparison of the two systems. The means of supply of fresh warm air in these two buildings, and of removing the air made impure by exhalations, are somewhat peculiar, and merit examination.

With regard to the architectural design and external appearance of the buildings, and the laying out and ornamentation of the grounds, I can only say that you must see and judge for yourselves whether Mr. Hopkins's wish that they should be an ornament to the city has been successfully complied with. So far as external ornamentation is concerned, it is confined almost entirely to the large buildings on the west, or Broadway, front, which it was felt should harmonise in style of decoration. These central buildings, consisting of the administration, with the one pay ward on either side, are constructed of pressed brick with ornamentation of a dark blue, fine-grained, hard, and durable stone, known as Cheat River stone, and of moulded terra-cotta of the colour of the brick. The external designs for these, as for all the other buildings, were furnished by Messrs. Cabot and Chandler, of Boston, and I think we have good reason to be well satisfied with the results they have produced. The grounds are laid out and planted in accordance with designs furnished by Mr. E. W. Bowditch, of Boston.

As regards construction, I do not hesitate to affirm that these are the best built buildings of their kind in the world. The material is the best, the most skilled and careful workmen were employed, and, above all, the work received the most careful, conscientious, and intelligent supervision as it progressed. For this supervision we are indebted to Mr. John Marshall in the beginning, and to Mr. William H. Leeke for the remainder and conclusion of the work; and we are also indebted to the latter for many valuable suggestions as to modes and details of finish, which are so important in a hospital. The details of the complicated and extended system of heating, ventilation, and plumbing were designed and the work executed by Messrs. Bartlett, Hayward, and Co., of this city. I should like to go on and mention a number of other names of persons who have done good work here, but want of time forbids. I will only say that these buildings embody the counsels and suggestions of many men and women in this country and abroad, but among them all there is no one who, from the very beginning of the conception of the idea of this magnificent gift in the mind of Johns Hopkins down to this present moment, who has had more to do with shaping the results, who has furnished more valuable suggestions, who is more thoroughly acquainted with all that has been done, and why it has been done, who has worked so unselfishly, and who more deserves honour in this connection, than the president of the board and chairman of the building committee through the whole progress of the work, Mr. Francis T. King.

Briefly and incompletely as I have sketched these salient points of the plans and purposes of this hospital, I hope I have, nevertheless, shown you that it is intended for other purposes beside providing shelter, food, and drugs for the sick. In saying this I have not the least wish to undervalue or disparage those institutions which do make this their main or only object. There is abundant need of their existence and work also; but this institution should not be judged by the rules which apply to them; it cannot be managed after their fashion; if it does not produce results different from theirs, it is a failure, and the expenditure upon it a mistake.

Thus far I have been speaking of the buildings only, and trying to give you some idea of the motives which led to their being as they are, and what they are, and not otherwise. From the beginning, however, it has been recognised that the buildings and machinery are only means to an end, tools which must be handled by skilled workmen to produce the desired results; and throughout all these years of planning and building, the question of organisation and of the sort of men and women who were to use and work with these things have not been lost sight of. It is true that no attempts were made to select and engage individual members of the hospital staff until quite recently; but there was, nevertheless, a tolerably definite conception as to the ideas, mode of work, character, and wants of those who are to constitute this staff, and when the time came for selecting it was made by this standard.

(*To be continued.*)

* An address delivered at the opening of the hospital, May 7, 1889.

